# Multiple Shocks, Coping and Welfare Consequences: Natural Disasters and Health Shocks in the Indian Sundarbans

**DOI:** 10.1371/journal.pone.0105427

**Published:** 2014-08-29

**Authors:** Sumit Mazumdar, Papiya Guha Mazumdar, Barun Kanjilal, Prashant Kumar Singh

**Affiliations:** 1 Population Health & Nutrition Research Program (PHN-RP), Institute for Human Development, New Delhi, India; 2 Department of Policy Studies, TERI University, New Delhi, India; 3 Future Health Systems–RPC India, and Indian Institute of Health Management Research, Jaipur, India; Rajarata Univeresity of Sri Lanka, Sri Lanka

## Abstract

**Background:**

Based on a household survey in Indian Sundarbans hit by tropical cyclone *Aila* in May 2009, this study tests for evidence and argues that health and climatic shocks are essentially linked forming a continuum and with exposure to a marginal one, coping mechanisms and welfare outcomes triggered in the response is significantly affected.

**Data & Methods:**

The data for this study is based on a cross-sectional household survey carried out during June 2010. The survey was aimed to assess the impact of cyclone *Aila* on households and consequent coping mechanisms in three of the worst-affected blocks (a sub-district administrative unit), viz. Hingalganj, Gosaba and Patharpratima. The survey covered 809 individuals from 179 households, cross cutting age and gender. A separate module on health-seeking behaviour serves as the information source of health shocks defined as illness episodes (ambulatory or hospitalized) experienced by household members.

**Key findings:**

Finding reveals that over half of the households (54%) consider that *Aila* has dealt a high, damaging impact on their household assets. Result further shows deterioration of health status in the period following the incidence of *Aila*. Finding suggests having suffered multiple shocks increases the number of adverse welfare outcomes by 55%. Whereas, suffering either from the climatic shock (33%) or the health shock (25%) alone increases such risks by a much lesser extent. The multiple-shock households face a significantly higher degree of difficulty to finance expenses arising out of health shocks, as opposed to their counterparts facing only the health shock. Further, these households are more likely to finance the expenses through informal loans and credit from acquaintances or moneylenders.

**Conclusion:**

This paper presented empirical evidence on how natural and health shocks mutually reinforce their resultant impact, making coping increasingly difficult and present significant risks of welfare loss, having short as well as long-run development manifestations.

## Introduction

Natural disasters are believed to have impacts affecting households in short, medium and long-term horizons. Apart from dealing a severe blow to household assets, income and livelihood streams, physical infrastructure and common property resources [1], [2], they run the risk of depleting human capital resources as well. However, it is believed that often the impact varies systematically across socio-economic groups, and the poor shoulder the disproportionate burden of the disasters in all its damaging consequences [2].

In particular, households in developing countries are often exposed to and struggle against a number of adverse events that disrupt income and consumption flows and are responsible for welfare losses [3], [4]. Unexpected and catastrophic shocks deplete household resources and lead to poverty traps [4], [5], besides deepening poverty among the already poor. Shocks invariably trigger coping measures as responses by the household, but the nature of the shock as well as form of the adopted coping strategies determine welfare consequences of the shocks. Research across developing world has documented a gamut of alternative coping strategies resorted to by households facing different shocks with the aim to maintain a smooth consumption flow [6], [7] and evade poverty traps [4]. However, there is little consensus on the success of these informal insurance mechanisms in smoothing consumption and prevent welfare losses [8]–[10]. The problem raises manifold in the event of aggregate shocks common to the community, as neighbourhood-network based informal support and risk-sharing after the incidence of the shock may become less commonly available [11]. Recent literature have documented however, that shocks typical to an individual household, rather than aggregate shocks are more difficult to insure due to higher magnitude and extent of impact; such shocks place a higher demand on the risk-coping mechanisms of the household [12]. It follows that a household naturally faces higher welfare risks if it has to face multiple shocks as the impact gets carried over and makes coping increasingly difficult.

Notwithstanding the rich literature in empirical development economics on the dynamics of shocks and its consequences across households, multi-shock studies are conspicuously rare. A few studies have considered multiple incidence of shocks households are exposed to, corresponding coping strategies and welfare consequences [12]–[14]. Nevertheless, most of the existing studies view the shocks, aggregate or idiosyncratic, in isolation as *discrete* events; we contend in this paper that shocks combine together to form a continuum and exposure to a particular shock significantly influencing coping mechanisms and welfare outcomes triggered in response to a subsequent shock. Which significantly influence coping mechanisms and welfare outcomes triggered in response in aftermath of a particular shock event. While this is much aligned to conventional wisdom, it has been rarely subjected to empirical investigation. To unpack the welfare consequences arising out of mutually reinforcing nature of shocks, we study the impact of idiosyncratic health shocks experienced by households during the year following a large climatic shock induced by a pre-monsoon cyclonic storm, cyclone *Aila* in Sundarbans delta in Bay of Bengal region during May 2009. The setting for the study is unique in itself: frequent exposure to natural inclemencies, common to other delta regions in South Asia, is most likely to induce alternative anticipatory strategies to diversify livelihood risks and prevent consumption shortfalls. On the other hand, considerable geographical barriers and poor infrastructure makes a trivial adverse event assume greater proportions and pose increased challenges to a household. Shocks and their outcomes in the context of Sundarbans, have various manifestations with contextual correlates often playing the key role.

We intend to posit the paper in the concerned literature from this perspective: It seeks to contribute to the empirical understanding of *combined* effect of large covariate shocks followed by smaller idiosyncratic shocks on households and understand how varying coping measures, influenced by shocks of reinforcing nature, determine welfare consequences. The major hypothesis we test is that *a large covariate shock, apart from its instantaneous impact, continues to influence and shape a household's behavioural responses in an extended time horizon; experiences of health or other individual shocks in this ensuing period further weakens coping ability and causes further welfare loss*. We however, do not attempt formal tests of consumption insurance but instead focus on more subjective and qualitative self-assessments by the household on the aggregate impact of multiple shocks.

Next, although climatic shocks in the form of natural disasters and extreme weather events are becoming more frequent worldwide and responsible for catastrophic consequences [15], changes in household behaviour in response to such disasters have received much less attention in the shocks-insurance literature with the possible exception of few studies [16]–[19]. This paper aims to bridge this gap and provide empirical evidence on welfare consequences of tropical cyclone *Aila* in Indian Sundarbans, as short and medium term development effects of climate-related shocks.

## Data and Settings

**Figure 1:**
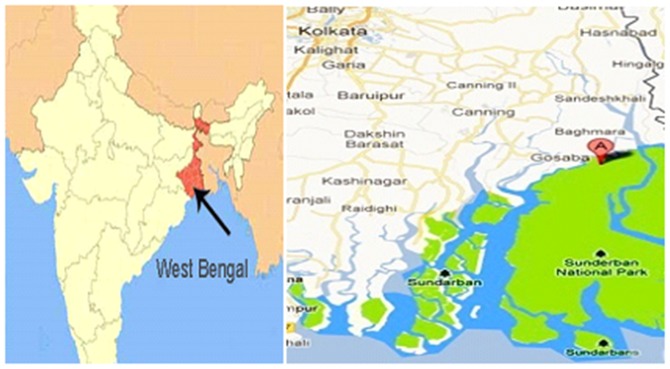
Study site: A mark on map (not on scale) depict the actual local of the study area, Sundarbans, West Bengal, India.

The Sundarbans, the world's largest riverine delta and one of the UNESCO global heritage sites, is a belt of mangrove forests and estuarine islands spreading through the extreme south of West Bengal, an eastern Indian state, and Bangladesh, the neighbouring country. The Indian part of the Sundarbans covers around 9630 square kilometres in West Bengal, spreading across 106 islands in 19 administrative blocks in two districts. As shown in the map (**Figure 1**), a large part of the Sundarbans (about 2600 sq. km) is protected as a reserve forest, also known as the Sundarbans Tiger Reserve. The area outside the reserve forest (54 islands), home of about 4 million people, is the human face of the Sundarbans. In sharp contrast to its natural face, the human face of the Sundarbans epitomizes abject poverty, deprivation and acute suffering. Due to harsh geographical challenges, the islanders struggle to survive on subsistence-level returns from diminishing natural endowments, depending almost entirely on rain-fed/mono-crop agriculture, the forest (for forest products) and the rivers/estuaries (for fishing) which hardly provide adequate support to the households in terms of income and employment. The extent of poverty can also be gauged by the fact that a little less than half the population (47%) belongs to the historically marginalized groups (such as scheduled castes and scheduled tribes) and more than half the farming community (55%) are landless labourers [20]. The issues related to biodiversity, ecological balance, and livelihoods in the Sundarbans are, however, dwarfed by a more serious threat which is generated by the global phenomenon of climatic change. Increasing height of sea-levels, due to global warming, has already led to disappearance of a few islands within the region and threatens to wipe out a large part of the Sundarbans in a few decades [21], [22]. Other environmental risks manifest in events such as sharp rise in water temperature [23], irregular rainfall, higher frequency of cyclones [24], rapid coastal erosion etc. considerably intensifies vulnerabilities of life and livelihood in low-lying deltaic regions. Sundarbans were hit by a devastating tropical cyclone – *Aila* – rampaging through the area on May 25, 2009. Within minutes, storm and consequent high tide wiped out a large part of river embankments, made thousands of villages disappear under water, killed hundreds of people, and rendered more than 400,000 homeless [25].

The data for this study is based on a cross-sectional household survey carried out in the area during June, 2010. The survey was aimed to assess the impact of cyclone *Aila* on households and consequent coping mechanisms in three of the worst-affected *blocks* (a sub-district administrative unit), viz. Hingalganj, Gosaba and Patharpratima. According to the official records of the Department of Planning and Development, Government of West Bengal these blocks were among the worst affected by the cyclone, experiencing near-devastation of crops across all the villages due to breaches of river embankment [26]. From each of these three blocks, two villages were selected purposively. Since all the blocks were universally hit by the cyclone, geographic representativeness formed the foremost consideration while selecting the villages. The survey was then conducted in 30 households chosen from each of the six study villages, following systematic sampling method. The survey covered 809 individuals from 179 households, cross cutting age and gender. A separate module on health-seeking behaviour served as the information source of health shocks defined as illness episodes (ambulatory or hospitalized) experienced by household members.

The survey had no direct questions to ascertain household consumption or income. Instead a more qualitative approach was followed to gauge welfare consequences of different shocks, self-assessment of the impact caused by these sudden and extreme events and alternative coping measures employed. We describe these in detail while elaborating on the focal points of the paper below.

We start with exploring the consequences of and coping measures employed against cyclone *Aila* by the study households. Drawing on the survey data, we try to understand

- *How do people respond to large, aggregate climatic shocks and natural disasters?* and,
- *Do they employ different coping mechanisms, depending on the extent of impact caused by the shock?*

Even for a covariate shock (such as a natural disaster), impacts on assets and livelihoods of households are rarely uniform and some households are more vulnerable to the effects of the shock, typically due to their low resource base and lower entitlements or reliance on risky livelihood strategies [9]. Both pre-existing vulnerabilities as well as extent of impact of such large shocks shape the coping strategies employed by the household. We hypothesize likewise that households experiencing a higher ‘relative’ extent of damage from cyclone *Aila*, as also the poor, less-educated households with less diversified livelihood strategies are more likely to employ ‘risky’ coping mechanisms – coping strategies that can have a detrimental impact on household well-being in latter periods.

To grade the extent of impact of *Aila* on households, members were asked about their self-assessment on the extent of impact *Aila* had on their assets, sources/means of livelihood and across their communities; we consider the self-assessed impact responses for household assets and consumption goods as the *impact* variable. The following questions were used to calculate the impact variable: a) Please rate the extent of damage (Devastating = 1, Moderate = 2, Partially/little affected = 3) caused by *Aila* on household assets/property such as homestead, plantations, food stocks, clothing etc. b) Please rate the extent of damage (Devastating = 1, Moderate = 2, Partially/little affected = 3) caused by *Aila* on the items comprising your/household's means of livelihood including soil fertility of farm lands, fishing, rearing livestock, hunting, business etc.

As stated above, the central aim of this paper is to test empirically whether, and to what extent do shocks act in a mutually reinforcing manner, and shape resultant coping strategies and welfare outcomes. In doing so we pose the main research question thus:

*Do multiple shocks have a reinforcing effect and lead to adverse welfare consequences in Indian Sundarbans? Specifically, do households experiencing health shocks, subsequent to the aggregate climatic shock, face higher risks of welfare loss?*

Adverse climatic shocks in the form of natural disasters such as the cyclone *Aila* often have a long-run impact weakening a household's ability to withstand future, and often trivial, smaller individual shocks like illness of household members [1]. We examine whether experiencing such health shocks in the ensuing period post-*Aila* lead to significant welfare loss involving consumption shortfalls, school dropouts, postponement of marriage decisions and other social commitments, reduced savings etc. We employ alternative forms of the health shock variable as,

- an indicator variable denoting whether the head of the household and/or the spouse suffered from any illness during 30 days prior to the survey, and alternatively,
- whether any adult member of the household in the economically active age-groups have suffered from illness episodes.

While these were used as *moderate* forms of health shock, similar to the variables employed by Gertler and Gruber in 2002 [27], for a more severe health shock we employ a dummy for households denoting whether any member was hospitalized (except due to childbirth) during the last year.

### Ethics Statement

The study protocols and tools were approved by the Institutional Review Board (IRB) at Institute of Health Management Research, Jaipur, India. Before starting the survey, informed consent was obtained from adult respondents, mostly in the form of verbal consent. Written consent – such as signed affirmation to the informed consent statement – could be only obtained when the respondent could read the statement (in local language, Bengali) and sign his/her name in approval.

In the case of minors/children, proxy responses were collected from mothers in most of the cases and primary caregivers, where mothers were absent or dead. Similar to the rest of the information, verbal or written consent was obtained depending on the functional literacy status of the respondent and/or the mother/caregiver in question. These approaches were included in the statement accompanying the study protocol put up for approval by the IRB, and ethical approval for the same duly obtained before collection of data/conducting the interviews.

## Results and Discussion

### Impact of Climatic Shock and Coping Measures

Finding shows that out of the 180 households covered in the survey, 82 (46%) were officially classified as poor, possessing a government-provided below-poverty line (BPL) card that entitles access (mostly at subsidized prices or for free) to certain basic public utilities (for e.g. employment benefit programmes, subsidized food-grains, low-cost medical facilities etc.) to the holder. However, there is considerable scepticism regarding proper identification of a deserving BPL household, and it is widely believed that the benefits are misappropriated and identification of BPL households largely erroneous.

Although, survey had no direct questions on consumption expenditure or income, we had some qualitative information on economic status of the household. More precisely, the survey collected information such as: whether every member of the households had enough/to the stomach's fill/in adequate quantity (two-square meals) to eat in the last week? In response to this, households that reported ‘never had two-square meals’ and ‘had two-square meals occasionally/for few days’ during last week were classified as poor in the analysis. This measure can be regarded as an approximation of the *transient* poverty status of the household. According to the response to this question, we find 35% of the households as poor, and use this classification for further analysis. Additionally, we use a composite index of *permanent* household wealth (including household assets, land and livestock ownership, type of house, source of drinking water and toilet) similar to the index used by the Demographic and Health Surveys (DHS) studies in developing countries [28]. Both this and the subjective *transient* poverty indicator were found to be conforming: 48% of the households classified in the poorest one-third, were also classified as poor according to the subjective indicator.

In the study population, agriculture, either in self-owned plots or as share-croppers, were the predominant sources of livelihood, closely followed by daily wage-earners. We also tried to list occupation of each adult/economically-active household members, which was used to classify households into categories – whether livelihood practices/occupational patterns were diversified (with household members engaged in different types of occupation). About 65% of the households had non-diversified livelihood, which, intensifies risks of insuring consumption patterns in the face of unanticipated shocks.

An examination of the basic descriptives regarding the impact of shocks and coping measures adopted further reveals that 54% of the households consider that *Aila* has dealt a high, damaging impact on their assets – homestead, crops, livestock, food, clothing, tools and implements etc. For brevity, we refer to this group as *high-impact* households throughout the paper. Of these households, slightly more than one-third (∼35%) were poor, both by transient and chronic poverty standards. Apparently, the *a priori* impact of *Aila* does not seem to be disproportionately harsh on the poor.

**Figure 2:**
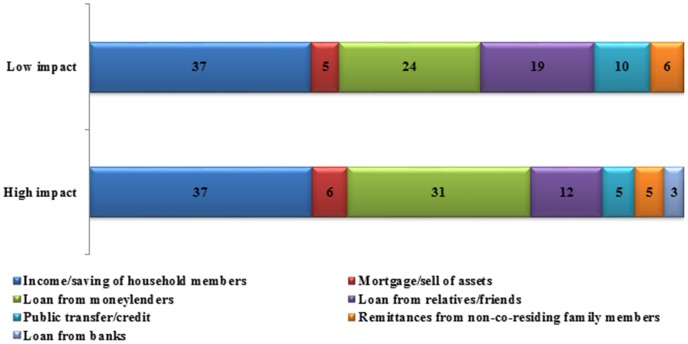
Percentage distribution of coping strategies against shock inflinted by cyclone *Aila*, according to perceived severity of the shock, Sundarbans, West Bengal, India.

However, most of these households report to have recovered from the effects of the shock at the time of our survey (about a year hence) by employing alternative coping strategies. A majority of the households irrespective of the extent of (self-assessed) damage caused by *Aila*, does not seem to employ a mix of coping strategies (mean number of coping strategies = 1.57). Common coping measures include informal credit from moneylenders (42%) or from relatives and acquaintances (23%), or falling back upon income or past savings of household members (57%). Public insurance in the form of government relief and aid (10%) were relatively rare – institutional credit from banks were hardly accessed (less than 3%) – as was mortgage or distress pawning of assets (8%) (**Table1**). Taken together, 48% of the high-impact households had resorted to drastic form of coping measures such as mortgaging of remaining assets or seeking informal credit from moneylenders (likely to be offered at usurious rates) which affects households' living standards in the longer run by placing future demands for financing loans. Again, such form of coping does not seem to correlate straightforwardly with poverty status, although a little more than half the poor households were found to resort to such coping measures, irrespective of the impact of *Aila*. If we ignore the role of self-insurance out of income and/or past savings, **Figure 2** further suggests that high-impact households tend to rely more (compared to the lesser-impacted households) on loans from moneylenders and mortgage or selling assets, both being risky coping measures with the potential to affect household consumption streams and living standards in an extended time horizon. Further, the high-impact households also appear to gain lesser from extended familial or societal networks or from public transfers/aids and thus might find smoothing consumption difficult. Again, similar to the perceived severity of the impact of *Aila*, coping patterns of the poor were largely found similar to that of non-poor households, within the high-impact category.

**Table 1:** Average proportion of households employing alternative coping strategies in response to climatic shock caused by cyclone *Aila*, Sundarbans, West Bengal, India.

Coping mechanisms against shock rendered by Aila	Lower impact of Aila	Higher impact of Aila	Total Sample
Income/savings of household members	57.32	56.70	56.98
Mortgage/selling assets	7.32	9.28	8.38
Loan from moneylenders	36.59	46.39	41.90
Loan from relatives/friends	29.27	18.56	23.46
Public transfers/credit (including help from NGOs/SHGs/CBOs)	14.63	8.25	11.17
Remittances from non-co-residing family members	8.54	7.22	7.82
Loan from banks	-	5.15	2.79

To gain further evidence on the influence of *pre-shock* vulnerabilities or household attributes on the choice of coping measures, we run a series of logit regressions. Specifically we estimate adoption of coping strategy *S_ij_* for the category of perceived severity of the climatic shock *H_j_* (i.e. whether the household is high-impact household, or otherwise), controlling for a vector of pre-shock vulnerabilities *M_i_* (for e.g. household wealth and poverty status, education and age of the household head, household size, ethnicity, occupational diversity) and village-level fixed effects *V_k_*. Or, 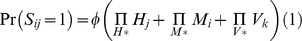



where subscripts *'i'* (i = 1,2.....n) denote individual-level attributes, *'j'* (j = 1,2,....n) denote type of shocks and *'k'* stands for villages (k = 1,2,3,4,5,6, for each of the sample villages); 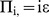
{H,M,V), indicates the vector of parameters to be estimated. Of our particular interest is to test the null hypothesis that belonging to a high-impact household, conditional on controlling for other pre-shock vulnerabilities and village-level fixed effects, does not lend towards adoption of a particular form of coping strategy, i.e.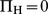. We estimate the models using logistic regression and report the odds-ratios in **Table 2**. Results indicate that high-impact households are significantly less likely to receive loans from relatives and other societal networks or support from public transfers or other institutional help to cope against the impact of the shock dealt by *Aila*. However, statistical evidence falls short of suggesting that they are significantly more prone, compared to the lesser-affected households, to opt for mortgaging assets or seek loans from moneylenders, as also for both actions jointly.

**Table 2:** Effect of climate shock intensity on use of different coping strategies, Sundarbans, West Bengal, India.

Coping Strategies	Odds Ratios	Standard Error	Pseudo - R2	N
Income/savings of household members	0.954	−0.31	0.054	179
Mortgage/selling assets	1.858	−1.234	0.2137	151
Loan from moneylenders	1.521	−0.523	0.1243	179
Loan from relatives/friends	0.501**	−0.198	0.0943	164
Public transfers/credit (includes support from NGO/CBO/SHGs)	0.327**	−0.183	0.2338	164
Remittances from non-co-residing family members	0.75	−0.468	0.1187	149
Risky coping strategies^#^	1.282	−0.425	0.0898	179

Note: ^#^Includes both ‘moneylenders’ and ‘mortgage etc.’ as coping strategies.

Second column values are odds ratios from logit regressions on the predictor variable denoting whether the household had suffered relatively greater impact from Aila. The indicator variable is based on a self-rating question on impact of *Aila* on eight categories of household assets and means of livelihood with a household reporting ‘devastating’ for more than half the categories classified as of suffering a greater impact – termed as ‘high impact’ households. The logit model additionally controls for *pre-shock* vulnerabilities (see text) and village-level fixed effects. The t-statistic tests for the hypothesis that the variable is not different from zero. * p<0.1, ** p<0.05.

### Health Shocks – 'Health' Effects of Climatic Shocks?

Before we proceed to link up and analyse the resultant impact of health shocks following the climatic shock inflicted by *Aila*, this section briefly takes up the question: Are there any *direct* ‘health’ effects of climatic shocks? In other words, do households, reporting to have experienced more severe forms of climatic shock also more likely to experience a negative change in health status in the aftermath of the climatic shock?

The evidence emanating from the literature is largely inconclusive: Some studies have found little or no evidence of associating outbreak of epidemics directly with natural disasters [29], [30], but to an extent with pre-existing poor sanitary conditions or large-scale displacements caused by the disaster [31]. On the other hand a number of reports [32] suggest otherwise – for instance a study [33] observed outbreaks of infectious diseases more common across the developing world following large-scale tropical cyclones or report an increase in risk of morbidity among children [34] following such hazards. Apart from the immediate epidemiologic consequences disasters are also believed to cause unfavourable nutritional outcomes among children [35] or lead to lower healthcare utilization [2] through indirect pathways like reduced food availability and consumption shortfalls.

Adopting a different approach, here we examine whether people's assessments of health status systematically vary according to the relative intensity of the climatic shock faced by the household. The survey asked respondents to rate the health status of all household members in terms of six age-groups (60+ adults by gender, 15–59 adults by gender, 6–15 children and less than 5 children) in a pre/post-shock comparative design on a scale of 1–3, with 1 as a positive change and 3 as negative. We calculated average health indices from the responses as a dummy for each age-group rating for whether a negative change was experienced post-*Aila* and then averaging ‘scores’ for the household, apart from such indicator variables for each age-sex groups. **Table 3** presents basic correlations and statistical test of means for relative impact of the climatic shock and perceived changes in overall health status of household members. The null hypothesis we test for rejection is that perceived changes in health status do not vary significantly between high impact and lesser impact households.

**Table 3:** Percentage of households with members in different age-sex groups reporting a deterioration of self-assessed health in the one year following *Aila*, Sundarbans, West Bengal, India.

Health impacts	Child (0–5 yrs)	Child (6–15 yrs)	Adult male (15–59 yrs)	Adult female (15–59 yrs)	All adults (15–59 yrs)	Elderly male (60+ yrs)	Elderly female (60+ yrs)	All elderly (60+ yrs)
Low-impact	9.76	23.17	45.12	43.90	56.10	17.07	9.76	20.73
High-impact	22.68	31.96	52.58	55.67	65.98	16.49	14.43	23.71
Combined Sample	16.76	27.93	49.16	50.28	61.45	16.76	12.29	22.35
Difference in Means	−12.92	−8.79	−7.46	−11.77	−9.88	0.58	−4.68	−2.98
*t-statistic*	*−2.328* **	*−1.305* *	*−0.991*	*−1.571* *	*−1.353* *	*0.103*	*−0.947*	*−0.474*

*p<0.1,

** p<0.05,

*** p<0.001.

As seen from **Table 3**, adult household members and children in the age-group of 6–15 years are more susceptible to experience a deterioration of health status in the period following the incidence of *Aila*. Close to two-third of the households reported witnessing a fall in health-status of an average member (standardized for age and sex). However, the difference in the proportions between households according to the self-assessed impact of the cyclone is significant only for children below 5 years and to an extent, among adult female members. Such a gradient of a higher likelihood of a child below 5 years of age from a high-impact household experiencing a negative health effect of the climatic shock is confirmed on further multivariate analysis (results not reported), although the same could not be indicated for other demographic groups of household members.

Although we have not investigated occurrence of specific health shocks (for e.g. incidence of communicable diseases) as epidemiological consequences of *Aila*, or attempt straightforwardly testing such causality, we do have sufficient evidence in support of a strong negative health effect of *Aila*. That such effect is significantly higher among children of households facing a greater impact of the climatic shock, supports the presumption that the extent of damage caused by climatic shocks such as *Aila* continues to negatively influence health status of vulnerable population groups such as children, and hence, can be expected to affect human capital in an extended time-horizon. While the results may suffer from a possible selectivity bias arising out of a largely purposive sampling and/or a natural control, this further refines indicatively the contention of Fuentes-Nieva and Seck [1]. We consider this aspect of welfare consequences arising out of differential impact of a climatic shock, where we introduce and test for an intensification effect attributed to smaller, idiosyncratic health shocks following cyclone *Aila* in the Sundarbans.

### Multiple shocks and impacts on well-being

Again, as in the previous section we start with examining the basic patterns of welfare outcomes and apply standard t-tests for difference in means across high and low-impact households. The welfare measures we consider involves wide-ranging domains viz., consumption, education, health, social commitments and future adaptive capacity and any amount of forced sacrifice or surrender in these domains were considered tantamount to welfare loss.

As it can be seen from **Table 4**, sacrifice of food consumption was nearly endemic in the period following the climatic shock, with postponing medical treatment of household members and forced dropout of children from schooling the other common consequences. Mostly due to higher volatility of incomes induced by transitory livelihood patterns after *Aila*, and meeting consumption needs mostly through dissolving leads more than half the households to report significant depletion of household savings. Notably, for almost all the welfare-domains, high-impact households were the worse-hit. The difference in reported proportions incurring such welfare losses is highly significant in the case of school-dropouts or for discontinuation of children's education, postponing medical treatment of household members, or marriage decision of daughters and avoiding social commitments such as attending communal gatherings and religious celebrations. If we note in passing that the incidence of the climate shock was not particularly heavier on the poor (close to 65% of the worse-hit households in the survey were non-poor), it clearly follows that *Aila* has thrown open poverty traps in varied manifestations – short-run consumption sacrifices to adverse human development outcomes like forced dropouts from schools having long-run consequences – and also weakening future adaptive mechanisms. Also, it appears that social engagements like marriages and community interactions, viewed as informal insurance mechanisms against future shocks [36], [37] also run the risk of being weakened, if not damaged.

**Table 4:** Proportion of households incurring sacrifices of different items according to self-assessed intensity of climatic shock, Sundarbans, West Bengal, India.

Sacrifices made by the household in the year following cyclone *Aila*	Low Impact	High Impact	Total Sample	Diff. in means	t-statistic
Food consumption	84.15	91.75	88.27	−7.61	−1.5778*
Education of children	29.27	51.55	41.34	−22.28	−3.078***
Postponing daughter's marriage decisions	3.66	8.25	6.15	−4.59	−1.2724**
Medical treatment of household members	58.54	82.47	71.51	−23.94	−3.6449***
Social commitments and responsibilities	31.71	47.42	40.22	−15.72	−2.1521**
Purchase of luxury goods	37.80	39.18	38.55	−1.37	−0.1867
Purchase of house/other assets	21.95	23.71	22.91	−1.76	−0.2777
Savings	60.98	51.55	55.87	9.43	1.2644
Preparedness to future contingencies#	24.39	55.67	41.34	−31.28	−4.4387

# building embankments, dwelling repairs etc.

*p<0.1,

** p<0.05,

*** p<0.001.

However, while this suggests in the broad favour of the premise of a covariate shock having a significantly different and adverse welfare consequences for the relatively worse-affected households, this does not provide any answer that whether the *intensification-effect* of smaller, accompanying idiosyncratic shocks accentuate such risks, in the lines of the hypothesis we had set earlier. To allow for the effect of health shocks as possible intensifier and test whether experience and effect of shocks mutually reinforce and lead to higher risks of welfare loss, we run an initial set of logit models for each individual welfare item mentioned in **Table 4**.

We start with looking for any unadjusted effect of health shocks in the naïve models, assuming climate shock to be fully covariate and of having a homogeneous impact across households. We estimate thus:
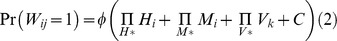



Where *W_ij_* is an indicator function of whether welfare loss (sacrifice or surrender of ‘consumption’) of any particular welfare item ‘*j*’ for the ‘*i*’th household, in the face of a health shock *H_i_*, *C* is the constant (or fully covariate) climate shock homogeneous in its impact across households and controlling for a vector of pre-shock vulnerabilities *M_i_* and village-level fixed effects *V_k_* as in Equation (1) above. 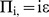 {H, M, V}, indicates the vector of parameters to be estimated. We test for the rejection of the null 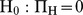, i.e. incidence of health shock does not cause an adverse outcome in *W_ij_*. As described previously, we have primarily considered self-reported illness (during 30 days prior to the survey) of the head of the household or the spouse as an indicator of health shock; a similar variable involving economically-active adult members was used as a sensitivity check for parameter estimates and reported episodes of hospitalization was considered as severe form of the health shock.

We have also computed an aggregate index of welfare loss using principal component analysis of the individual (normalized) welfare items stated above and retaining the first principal component, an approach again similar to the computation of ‘wealth’ index [28] in DHS and considered apt for survey data where income or consumption expenditure is absent [38], [39]. The reliability coefficient of the composite scale was tested and found to be satisfactory (Chronbach's α = 0. 6591). We estimate hence, a variant of (2) above, as a conventional OLS regression, and test for 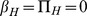:
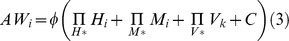
If we introduce the differential impact of the climatic shock in the model above (3) our estimation problem assumes the following form:
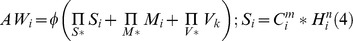



where *S_i_* is an interacted (multiple) shock variable; 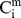
indicates incidence of climatic shock with alternative outcomes ‘m’, with

m = 1 if household i is a high-impact household

 = 0; otherwise

and similarly for health shock indicator, 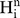, where

n = 1 if household i experiences a health shock

 = 0; otherwise

Equation (4) is estimated as conventional OLS, with an interaction term denoting the multiple shock variable *S_i_*, leading to classify households in four mutually exclusive groups, viz. those experiencing a lower impact of climatic shock and no health shock (the omitted reference group), high impact households from climatic shock but not experiencing any health shock and its complement households which have experienced health shocks but a lower impact of climatic shock and, finally the worst-affected households having experienced both health shocks as well as a relatively greater impact of the climatic shock. The last category is our main group of interest, and we test thus as null,
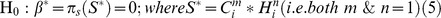



Parameter estimates of (2) yield little suggestive evidence (not reported in **Table 5**): the only items for which occurrence of a health shock was associated with a significantly higher likelihood of welfare loss were for purchase of luxury items/consumer durables or houses and other fixed assets. Moving towards estimating (3) yields interesting results. Ignoring restrictively the differential impact of climatic shock across households, we find that households experiencing a health shock, both in terms of illness of household head/spouse or other economically active adult members, are also likely to experience considerable aggregate welfare loss (about 40% higher than households not experiencing such a shock). This however, does not allow for a differential pre-disposing impact of the climatic shock, and consider the effect of health shock as an intensifier, which, is our major question of interest. This is attained by estimating (4).

**Table 5:** Multiple Shocks and Aggregate Welfare Losses due to cyclone *Aila*, Sundarbans, West Bengal, India.

Panel AOLS Regression Model for Aggregate Welfare Consequences of Health Shocks			
Health Shock Parameters (Predictor Variables)	β	R^2^	Adj. R^2^
*Outcome: Aggregate welfare consequences (in a continuous scale)*			
Health shocks (moderate) - illness of household head/spouse	0.433**	0.124	0.037
	(−0.154)		
Health shocks (moderate) - illness of any economically active household member	0.481**	0.131	0.045
	(−0.157)		
Health shocks (severe) - hospitalization of any household member	−0.096	0.082	−0.008
	(−0.174)		

Panel BOLS Regression Model for Aggregate Welfare Consequences of Multiple Shocks			
Interacted Shock Parameters (Predictor Variables)	β	R^2^	Adj. R^2^
*Outcome: Aggregate welfare consequences (in a continuous scale)*			
Health shock (moderate) alone	0.527**	0.1646	0.0706
	−0.222		
High-Impact Climatic Shock alone	0.480**		
	−0.217		
Both High-Impact Climatic Shock and Health Shock (moderate)	0.874***		
	−0.218		

Panel CPoisson Regression Models for Count of Adverse Welfare Outcomes			
Interacted Shock Parameters (Predictor Variables)	Coefficient	% change in expected count of Y for discrete change in X	Marginal change in expected count of Y for discrete change in X
*Outcome: Count of Adverse welfare outcomes*			
Health shock (moderate) alone	0.223*	25	0.8872
	−0.124		
High-Impact Climatic Shock alone	0.288**	33.3	1.1451
	−0.119		
Both High-Impact Climatic Shock and Health Shock (moderate)	0.438***	55	1.7448
	−0.116		

*p<0.1,

** p<0.05,

*** p<0.001.

The results, convincingly rejects the null hypothesis set in (5) and strongly supports the argument that joint occurrence of both a high-impact climatic shock followed by a health shock significantly increases the risk of aggregate welfare loss (by about 87%), as opposed to households either experiencing none of the shocks, or any one of the two types of shocks considered. A similar inference emerge when we consider the health shock variable involving any economically active household member, and not just the household head/spouse alone. However, for severe forms of the health shock in the form of hospitalized episodes, such conclusions do not necessarily follow.

If aggregate welfare, which in our exercise is a composite index of a number of individual welfare domains, is highly susceptible to witness a sharp fall following multiple incidence of shocks, it is of interest to examine whether any particular domain of welfare exhibit a higher relative risk. To examine thus, we run a series of logit regressions using the same set and form of predictors as in (4), but having an indicator variable for each welfare item as the outcome variable. Results from the estimated models are depicted in the form of the strength of statistical significance of odds ratios from logistic regression outputs for each welfare items, and interaction groups (**Table 6**). From the last column to the right, it is evident that multiple shocks are particularly responsible in postponing marriage decisions, and inhibiting resilient measures against future climatic shocks, and to a lesser extent in increasing the likelihood of discontinuing children's education and disrupt non-food consumption expenditure such as purchasing assets and other items of daily use.

**Table 6:** Significance of Multiple Shocks on Individual Items of Welfare, Sundarbans, West Bengal, India.

Domains of Welfare Loss/Sacrifice/Surrender of 'Consumption'	Both High-Impact Climatic Shock and Health Shock
Food consumption	No-impact
Education of children	Moderate
Postponing daughter's marriage decisions	High
Medical treatment of household members	No-impact
Social commitments and responsibilities	Low
Purchase of luxury goods	Low
Purchase of house/other assets	Moderate
Savings	No-impact
Preparedness to future contingencies^#^	High

It is however, difficult to quantify and compare across households the extent of aggregate welfare loss, more so when the welfare dimension lacks any quantitative data. In other words, while we find above that households experiencing multiple shocks are at considerable risk to experience a higher loss of aggregate welfare, and face adverse consequences on a number of welfare domains, the results fall short of providing a quantitative estimate of the *degree* of such adverse welfare consequences. An alternative is to compare the number of welfare items for which household members report a forced sacrifice or reduced ‘consumption’ and, model the number of such welfare outcomes as conventional count data models.

An average household in our study sample suffers welfare loss in almost four items of welfare (average = 4.06); the difference in means between households experiencing multiple shocks (average = 4.87) and the rest (average = 3.75) is highly significant (t-statistic = −3.113). For a more conclusive inference, we estimated a Poisson's regression model using the previously explained interacted shock variable as the predictor of interest, and with other applicable controls. We exploit the ‘listcoef’ and ‘prchange’ routines in STATA version 10 [40] provided by Long and Freese [42], to derive a quantitative estimate of the likelihood of witnessing welfare loss on additional items by multiple shock households vis-à-vis other groups of households in the interacted shock variable used in equation (5) above. The ‘listcoef’ yields the percentage change in the expected count of y (the count of adverse welfare outcomes) holding other variables constant; prchange computes discrete change, or marginal effects in the expected count for a change in the interacted shock variable from the base group 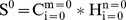 (i.e. reference group experiencing less-impact of climatic shock C and no health shock H) to the multiple shock category 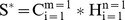. Results are shown in the lower panel of **Table 5**.

The results reiterate the findings earlier from the OLS models for aggregate welfare: Having suffered multiple shocks increases the number of adverse welfare outcomes by 55%, holding all other background attributes constant. Whereas, for suffering either (high-impact of) the climatic shock (33%) or the health shock (25%) alone increases such risks by a much lesser extent. Similarly, experiencing multiple shocks increase the expected adverse welfare consequences by an additional 1.75 welfare items, as against 1.14 and 0.88 items for the two other single-shock comparison group mentioned above.

### Multiple Shocks and Coping Strategies

An indirect way of examining how shocks are interlinked in their resultant impacts on households, and that being hard-hit from a preceding shock can actually cause coping with subsequent shocks more difficult can be tested by looking at the coping mechanisms employed by households in response to the latter shock. The survey in *Aila*-affected Sundarbans asked respondents on the different coping mechanisms used in the event of illnesses, requiring ambulatory or hospitalized care. The focus was specifically on the coping mechanisms as means to finance the cost of treatment or allied expenses in the year following *Aila*. Households were asked to assess the *difficulty* faced in such financing pattern as compared to the pre-*Aila* year on a 5-point scale with ‘1’ being ‘more difficult’ and ‘5’ being ‘much easier’. We converted the variable into a binary response indicating the incidence of difficulty (rating ‘1’) and used it as the main outcome variable. The analytical model was a simple logit regression to examine whether a household found it difficult to meet the ‘direct’ or immediate financial implications accompanying the health shock, given its relative experience of the larger climatic shock. In other words, we test whether, among all the households facing the health shock, those affected greater by the climate shock (‘high-impact’ households) are more likely to face such difficulty after standardizing for level of health expenses and other standard controls. We also examine whether these households (we refer these group as capturing the ‘joint shock’ dimension – high impact from climatic shock and experiencing health shock) are more likely to resort to debt-financing of health shock, or meet the expenses out of own income and savings. **Table 7** reports the parameter estimates from the models.

**Table 7:** Joint effect of climate and health shocks on coping strategies against expenditure following health shock, Sundarbans, West Bengal, India.

Coping against medical and non-medical expenditures following health shock^#^	Health Shocks alone (n = 45)	Multiple Shocks (n = 49)	Total Sample @	Diff. in Mean	t-statistic	Odds Ratios on Multiple Shock parameter∧	Pseudo R^2^
(A)	(B)	(C)	(D)	(E)	(F)	(G)	(H)
Difficulty in financing health expenses	53.3	83.7	69.1	−29.6***	−3.332	3.837** (2.471)	0.331
Financing through household income and/or dis-saving	68.9	48.9	58.5	20.0**	1.9769	0.263** (0.149)	0.196
Financing through informal debts and credit	35.6	59.2	47.9	−23.6**	−2.3324	4.623* (2.892)	0.344

Note: ^#^Expenditure items include direct costs of treatment, expenses on drugs and medicines, transport, and related expense such as those incurred on food or lodging for patients and accompanying person(s).

@ denotes that the testing of hypotheses for difference of means (results in column B-F) was carried out only for the households reporting an incidence of health shock (n = 94).

∧ The coefficients in column G are odds-ratios on the ‘multiple-shock’ variable – households experiencing both the health shock as well as high-impact due to climatic shock – from logit regressions with the dependent variable in the corresponding row of the first column. Health shock variable is for illness of household head and/or spouse. The comparison group for column G coefficients is households with health shock alone (with a less-impact of Climatic shock). Models additionally control for (log) total health expenses, *pre-shock* vulnerabilities (see text) and village-level fixed effects. Coping models (items B and C, in column A) also controls for the self-assessed ‘difficulty in financing’ variable.

Figures in parentheses are t-statistics testing for the hypothesis that the variable is not different from zero.

* *p<0.05, *** p<0.01.

Apart from the broad observations that the findings are much in accordance to the earlier finding for aggregate welfare consequences, with joint effect of both adverse climatic and health shocks significantly influencing coping measures, a few other interesting facts emerge. Firstly, multiple-shock households face a significantly higher degree of difficulty to finance expenses arising out of health shocks, as opposed to their counterparts facing only the health shock. Such gradient persists even after controlling for the level of healthcare expenditure incurred and other background attributes. Secondly, these households are more likely to finance the expenses through informal loans and credit from acquaintances or moneylenders, and less likely to manage such expenses out of their own income and savings. Thirdly and related, it may be important to observe the changing role of income and household savings as risk-coping mechanisms in the immediate aftermath of *Aila* and later, after the experience of health shocks between high-and less-impact households. While the difference in means for income and savings between these two groups of households was very thin and statistically insignificant, the divergence has sharply widened and assumed greater significance in the year since *Aila*. It appears hence, that while the less-impact households had largely recovered hence and were in a relatively better position to absorb the financial impact of health shocks through self-income, the high-impact households finds it increasingly difficult, most possibly having depleted household savings in post-*Aila* recovery and yet to smooth the income flow. Fourthly, as we have controlled in absolute terms for healthcare expenses, it seems that the major climatic shock has a pre-dispenser effect in increasing the propensity of the high-impact households to opt for more risky coping measures.

While a more formal test on decomposing welfare effects from a series of shocks a household experiences could have further clarified the relative importance of each type of shock on welfare outcomes, we have illustrated, through alternative tests and posing different questions, the strong possibilities of a smaller shock intensifying welfare consequences in the aftermath of a larger, aggregate shock, which was seen to have a significantly differential effect. One may also argue that a trivial idiosyncratic shock need not always have welfare implications, with the household able to smooth consumption and insulate well-being through alternative coping mechanisms. Along the same lines, our findings are indicative of how a devastative natural disasters or any other large covariate shocks, definitively weakens a households capacity to withstand even smaller idiosyncratic shocks like minor illnesses of household members, which in turn can have wider adverse welfare implications, or call for immediate, and often desperate measures to cope with multiple crises becoming of the shocks.

## Limitations of Present Study

This study has a few caveats that need to be acknowledged for better interpretation of the results, which lays out the assumptions we make in empirically testing the hypotheses for our sample. Firstly, and ideally such a test requires a strong *counterfactual* – what could have been the welfare consequences for households in the absence of a ‘predisposing’ covariate shock, or if the health shocks were experienced by households unaffected by the preceding natural disaster. The survey, a strict cross-section, had no such readily available comparison group. This pre-empt us to the alternative approach of considering the health shocks as an *intensifier* to the welfare consequences a household is likely to experience following the aggregate climatic shock, in a spirit similar to the analysis of treatment effects in the evaluation literature. In the survey, respondents were asked whether, in the year following the climatic shock the household had to postpone, sacrifice or surrender certain items (food consumption, education, healthcare, social commitments, marriage decisions, savings, acquiring assets and preparing for disasters such as building embankments, house repairs etc.) which we use to capture welfare loss. In doing so, we assume that the actions incorporate, apart from ex-post insurance measures against the climatic shocks, household responses to other shocks as well in the intervening period. The welfare loss measure, hence, is an aggregate of responses to *all* shocks faced by the household in the reference period, and not in response to a particular shock, the larger climatic shock in this case, alone. Although this assumption runs to the risk of ignoring other individual shocks (job loss, crime, interpersonal disputes, death etc.) and considering health shocks alone as the *intensifier*, we believe that doing so would not significantly over-estimate the impact of health shocks as previous multi-shock studies [12], [14] have found health shocks to be the predominant idiosyncratic shock affecting households. Moreover, we are more interested in the conceptual question of whether smaller idiosyncratic shocks reinforce or *intensify* adverse welfare outcomes following a large covariate shock, rather than on rigid quantification of welfare effects due to an accompanying health shock *per se*.

Secondly, apart from examining the consequences for each welfare items mentioned above, we observed the *aggregate* consequences as well. We recognize the possible theoretical problems of treating all the items equally. The composite welfare index we have used may also suffer from omissions of variables that may contribute to household's welfare. In this sense, our *non-income* welfare measure is incomplete, but similar to that used in qualitative analyses of poverty [42]. However, in the multivariate models to follow we control for aggregate ‘wealth’ reflecting a household's permanent income. This additionally allows us to have reduced-form specifications, and absolve from any possible bias arising out of having current or *transitory* income being endogenous. Thirdly, the sample, driven by the basic study objectives to assess household socio-economic conditions, health-seeking behaviour and coping mechanisms in the aftermath of the natural disaster, was largely purposive beyond the household-level (i.e. selection of administrative blocks and villages) and not having a sufficiently large size allowing rigorous quantitative models. Although we apply standard econometric tests of hypothesis to infer on results from the analytical models, a larger and more diverse sample would probably avoid Type I errors. With these qualifications in consideration, the results need to be interpreted with caution and best, if thought of in an indicative sense.

## Conclusions and Implications

In this paper we have argued and presented empirical evidence on how shocks mutually reinforce their resultant impact, making coping increasingly difficult and present significant risks of welfare loss, having short as well as long-run development manifestations. Several findings emerge from the results which add to the understanding of shocks and its impacts in developing countries. We contend that even a large, aggregate shock such as the tropical cyclone *Aila* is far from being truly homogeneous in its impact across households; Often the heterogeneity in the impact of such a large climatic shock works towards weakening the adaptive capacity of the households, and, as we have shown above, trivial idiosyncratic shocks like ill-health of household members in the ensuing period can cause significant welfare loss. Smaller individual shocks, as the findings suggest, contribute as intensifiers to wreck household consumption patterns and pose serious threats to maintain living standards, even when households struggle hard to recover from the impacts of the disaster. Although we recognize, and find support to similar findings elsewhere [12] of the predominance of health shocks in causing adverse welfare outcomes and less than complete smoothing of consumption patterns, it is evident that past experience of the climatic shock leads to higher difficulty to absorb the financial impacts arising out of health shocks and leads to significantly different, and often risky, coping strategies. Coping measures in themselves are rarely static; a household employing more stable coping mechanisms may be forced to settle down for ones more risky and carrying risks of future sacrifices, if shocks increase in its frequency and the resultant impact. Hence, it is imminent that in a setting where livelihood patterns are less diverse and formal credit markets marked by its poor reach, shocks, both sudden and catastrophic natural disasters or disease or ill-health have strong potential to disrupt consumption patterns and exert pressure to depress welfare outcomes.

Although we expect most of our findings to be relevant for any developing, agrarian settings where shocks are a way of life, Sundarbans has its own distinctive features that exacerbate welfare risks in the face of shocks. Widely exposed to frequent vagaries of nature, attributed much to the global pattern of climate change and heightened risks posed to life and livelihoods in low-lying, coastal regions [20], [43], development connotations in Sundarbans hangs on a precarious balance. In the lines of the emerging inferences from the present study, it may be gauged that frequent natural calamities such as floods and cyclonic storms, even at a much lesser scale may weaken adaptive capacities of households; disasters and other extreme climatic events hasten the breakdown of all coping mechanisms and push households further into chronic poverty traps. Often, it is the continuum of shocks that shapes a households welfare outcomes in geo-climatically challenged areas like the Sundarbans and explains the dynamics of coping mechanisms. Viewing shocks in isolation, even from a perspective of comparing and grading different types of shocks may lead to ignoring crucial dimensions of welfare and understand the myriad cycles of shocks, coping and its consequences in vulnerable societies. This paper is posited in this vacuum and highlights the need for more systematic studies in this area with further nuanced methodologies and study designs (longitudinal data - for example).

This paper suffers from a few limitations, though. Apart from the few qualifications we mention earlier, we are aware that most of the outcome measures, either for the impact of climatic shock, or forced sacrifice of ‘consumption’ on different welfare domains or items, or difficulty in meeting the financial demands arising out of healthcare expenses are based on subjective self-assessments of the respondent, and thus may be biased. However, increased reliance on such qualitative self-assessments can be noticed in contemporary development literature such as poverty and vulnerability assessments [42]. However, these measures are believed to work best when supplemented by quantitative evidence; we were plagued in this respect by absence of financial or living standards data. Nevertheless, since most of the results are found to be intact when alternative outcome or predictor variables were used, the inferences following are believed to be reasonably robust. Being a purposively designed study may also magnify some of the outcomes and deductions that follow; it is felt that this does not dilute the broad indications but argue in favour of further study to test the hypotheses more rigorously.

Few public policy imperatives are in order in the light of the contours of the findings of this paper. Firstly, a strong felt need is identified for instituting formal safety nets in climatically vulnerable localities in Sundarbans. This may involve reinforced embankments, storm centers, and houses that are more capable to withstand such extreme climate events. Ongoing government strategies (Government of West Bengal, undated) to mitigate adverse effects arising out of climate change and its accompanying weather events, must take cognizance of the interrelationships between vulnerability, risky livelihoods and resilient capacities of residents in the risky terrains of Sundarbans. Secondly, community-based action groups like Self-Help Groups (SHGs) or other Community-based Organizations may have an important role to play. Attempts to promote diversified livelihood practices, and encourage risk-pooling across communities through micro-finance and micro-insurance schemes and getting more communities involved is strongly called for. Evaluative research to better understand the efficacy of these safety nets to weather the multitude of shocks and risks facing the population of Sundarbans is also required. A more radical idea, as the last resort, may be to consider prospects of relocating people from high-risk coastal zones further inland, where climatic risks are less intense. Shocks and risks in Sundarbans are as sure as life itself. A concerted approach, both institutional and local-solutions oriented can help mitigate much of the risks and following its realization, the undesirable welfare outcomes in short as well as in the long run.
